# Long-term follow-up of comparative study of open and endoscopic lymphadenectomy in patients with penile carcinoma

**DOI:** 10.1007/s00464-023-10542-8

**Published:** 2023-11-10

**Authors:** Xue-Lu Zhou

**Affiliations:** Department of Surgery, Chashan Hospital of Guangdong Medical University, 92 Caihong Road, Chashan Town, Dongguan, 523127 Guangdong People’s Republic of China

**Keywords:** Penile carcinoma, Inguinal lymphadenectomy, Open, Endoscopy

## Abstract

**Background:**

Penile carcinoma is an uncommon cancer that develops in the penis tissue. The standard surgical method to manage regional lymph nodes after local excision is radical inguinal lymphadenectomy, but it has a high rate of complications. The objective of this retrospective study was to compare the long-term outcomes of endoscopic inguinal lymphadenectomy and open inguinal lymphadenectomy in patients with penile carcinoma.

**Methods:**

The study included patients diagnosed with penile carcinoma who underwent open inguinal lymphadenectomy (n = 23) or endoscopic inguinal lymphadenectomy (n = 27) at a single hospital between January 2013 and January 2021. Operation time, blood loss, drainage, hospital stay, postoperative complications, and survival rates were assessed and compared between the two groups.

**Results:**

The two groups were comparable in terms of age, tumor size and stage, inguinal lymph nodes, and follow-up. The endoscopic group had significantly lower blood loss (27.1 ± 1.5 ml vs 55.0 ± 2.7 ml, *P* < 0.05), shorter drainage time and hospital stay (4.7 ± 1.1 days vs 8.1 ± 2.2 days, and 13.4 ± 1.0 days vs 19 ± 2.0 days, respectively, *P* < 0.05), and longer operation time compared to the open group (82.2 ± 4.3 min in endoscopic group vs 53.1 ± 2.2 min in open group, *P* < 0.05). There were significant differences in the incidence of incisional infection, necrosis, and lymphorrhagia in both groups (4 vs 0, 4 vs 0, and 2 vs 0, respectively, *P* < 0.05). The inguinal lymph node harvested was comparable between the two groups. The mean follow-up time was similar for both groups (60.4 ± 7.7 m vs 59.8 ± 7.3 m), and the recurrence mortality rates were not significantly different.

**Conclusions:**

The study shows that both open and endoscopic methods work well for controlling penile carcinoma in the long term. But the endoscopic approach is better because it has fewer severe complications. So, the choice of surgery method might depend on factors like the surgeon’s experience, what they like, and what resources are available.

Penile carcinoma is a rare malignancy affecting the penis tissue, with a higher incidence rate in men over 50 years old and in developing countries. After local excision of penile cancer, radical inguinal lymphadenectomy has been the standard surgical approach for the management of regional lymph nodes, but it is associated with a high morbidity rate, with complications exceeding 50% [1, 2]. Although modified inguinal lymphadenectomy reduces the morbidity rate, it still ranges from 26.7 to 38.9% and carries a risk of false-negative histopathological results, which can compromise the oncological outcomes [3–6]. A novel approach, known as endoscopic subcutaneous modified inguinal lymphadenectomy has shown promising results in reducing morbidity without compromising the oncological outcomes [7, 8]. The choice of surgical approach should consider the extent of cancer and the surgeon’s expertise.

A long-term follow-up study that compares open and endoscopic lymphadenectomy in patients with penile carcinoma is essential in providing valuable insights into the efficacy, risks, and benefits of different treatment approaches. Additionally, such a study can help determine the long-term health outcomes and quality of life for patients, thus assisting clinicians and patients in making informed decisions about treatment options. Furthermore, the study’s findings can facilitate the development of new approaches to improve the care and support provided to patients with penile carcinoma. Therefore, this retrospective study aims to compare endoscopic and open inguinal lymphadenectomy in patients with penile carcinoma, assessing whether endoscopic lymphadenectomy is superior to open lymphadenectomy in terms of both oncological outcomes and postoperative complications during long-term follow-up.

## Materials and methods

### Study population and data selection

**Fig. 1:**
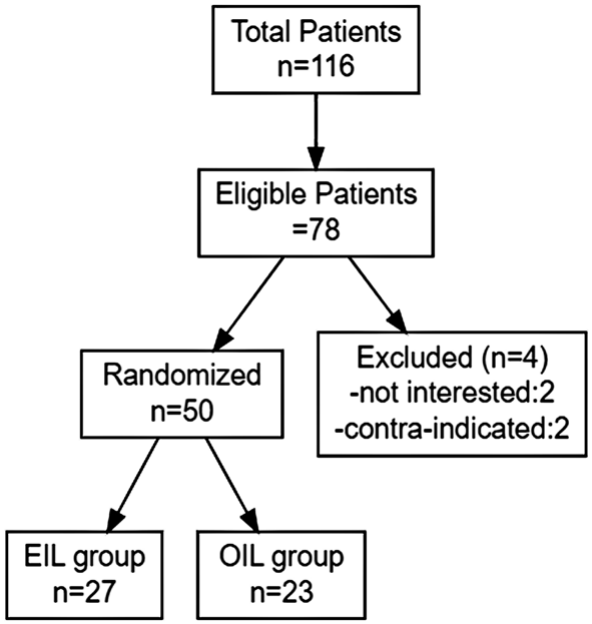
Patient flow chart for the clinical trial

This was a case–control study. The hospital’s database revealed a total of 116 patients who had been diagnosed and operated on for penile cancer between January 2013 and May 2021. Among these, 78 patients met the eligibility criteria, and we subsequently identified and included 50 patients in the study. Out of these 50 patients, 23 underwent an open approach, while 27 underwent an endoscopic approach, as illustrated in Fig. 1. The inclusion criteria were as follows: individuals under the age of 75, diagnosed with penile carcinoma without distant metastasis, able to undergo endoscopic surgery, and willing to provide informed consent. Patients with severe cardiopulmonary, liver, or kidney function insufficiency, significant coagulation dysfunction, distant metastasis, or those who withdrew from the research, were excluded from the study. The study received approval from the hospital’s ethics committee, and all patients provided informed consent.

The EIL group consisted of 27 patients with an average age of 57.7 years and an average BMI of 26.4. The average tumor size was 1.72 cm, with 13 patients classified as stage I and 14 as stage II. Of these patients, 26 underwent prophylactic bilateral EIL, and one received bilateral therapeutic EIL after a positive node biopsy. The OIL group comprised 23 patients with an average age of 58.2 years and an average BMI of 25.9. The average tumor size was 1.7 cm, with nine patients classified as stage I and 14 as stage II. All patients in the OIL group underwent bilateral OIL, and one received pelvic lymphadenectomy for metastases 1 year after OIL. The baseline data from both groups were comparable with no statistically significant difference (*P* > 0.05) (Table 1). All patients received prophylactic antibiotics half an hour before surgery, with 1 g of ceftriaxone administered intravenously.

**Table 1:** Patients’ characteristics of the two groups

Variable	OIL (n = 23)	EIL (n = 27)	*t*/χ^2^	*P*
Age (years)	58.2 ± 2.0	57.7 ± 2.2	0.35	0.76
Body mass index (BMI)	25.9 ± 1.8	26.4 ± 1.6	0.25	0.80
Tumor size (cm)	1.7 ± 0.1	1.72 ± 0.1	1.15	0.91
Tumor stage			0.20	0.65
T_1_N_0_M_0_ (stage I)	9	13		
T_2_N_1_M_0_ (stage II)	14	14		
Inguinal lymph nodes			0.27	0.60
Palpable	5	8		
Non-palpable	18	19		
Operation time (each side) (min)	53.1 ± 2.2	80.2 ± 4.3	5.30	0.00
Blood loss (ml)	50.0 ± 2.7	27.3 ± 1.5	8.41	0.00
Total number of lymph nodes harvested (average number/each)	536.6 (11.6 ± 2.9)	599.4 (11.1 ± 2.3)	0.08	0.78
Positive	16	18		
Negative	517.6	571.4		
Drainage duration (days)	8.1 ± 2.2	4.7 ± 1.1	1.60	0.01
Hospitalization (days)	19 ± 2.0	13.4 ± 1.0	3.50	0.00
Follow-up time (m)	60.4 ± 7.7	59.8 ± 7.3	0.79	0.45

### Surgical procedure

**Fig. 2:**
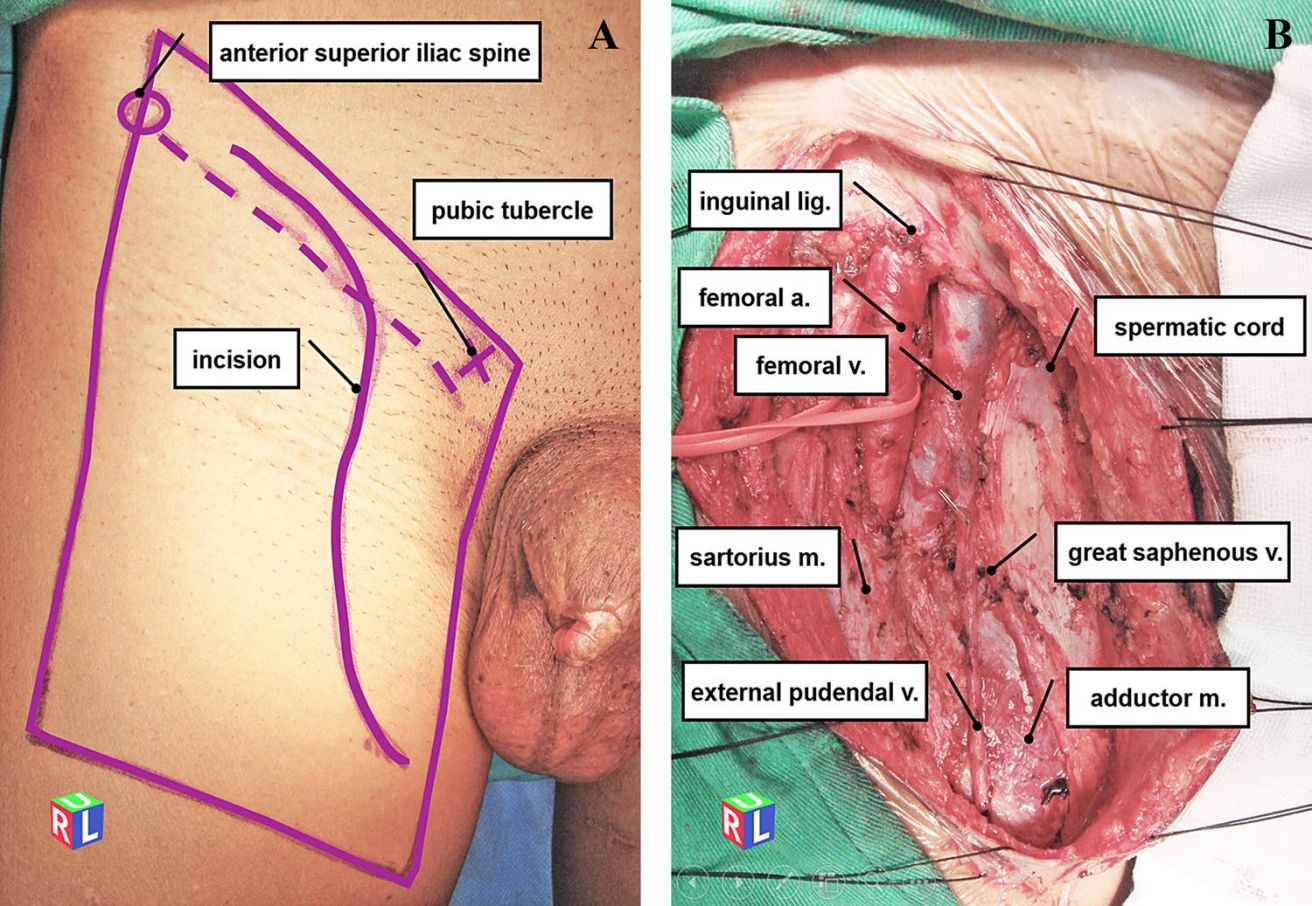
Modified open inguinal lymphadenectomy (right side) **A** Modified incision for inguinal lymphadenectomy. **B** Anatomical marks are clearly visible after radical dissection

Modified radical inguinal lymphadenectomy was performed for the OIL group. The procedure involved creating an S-shaped incision that began 2 cm medial to the anterior superior iliac spine and extended distally to the midpoint of the inguinal ligament. From there, the incision went down vertically along the projection line of the femoral artery and ended 3.5 cm inferior and medial to the fossa ovalis (Fig. 2A). The dissection boundaries were the apex of the femoral triangle inferiorly, the sartorius muscle laterally, the adductor longus muscle medially, and 10 mm superiorly to the inguinal ligament. Scarpa’s fascia was used to separate the skin flaps, and all subcutaneous lymphatic and adipose tissue were dissected. The saphenous vein was preserved as far as possible, and the deep inguinal lymph nodes were removed (Fig. 2B). Subcutaneous drainage was placed and maintained until drainage was less than 15 ml daily for 2 consecutive days.

**Fig. 3:**
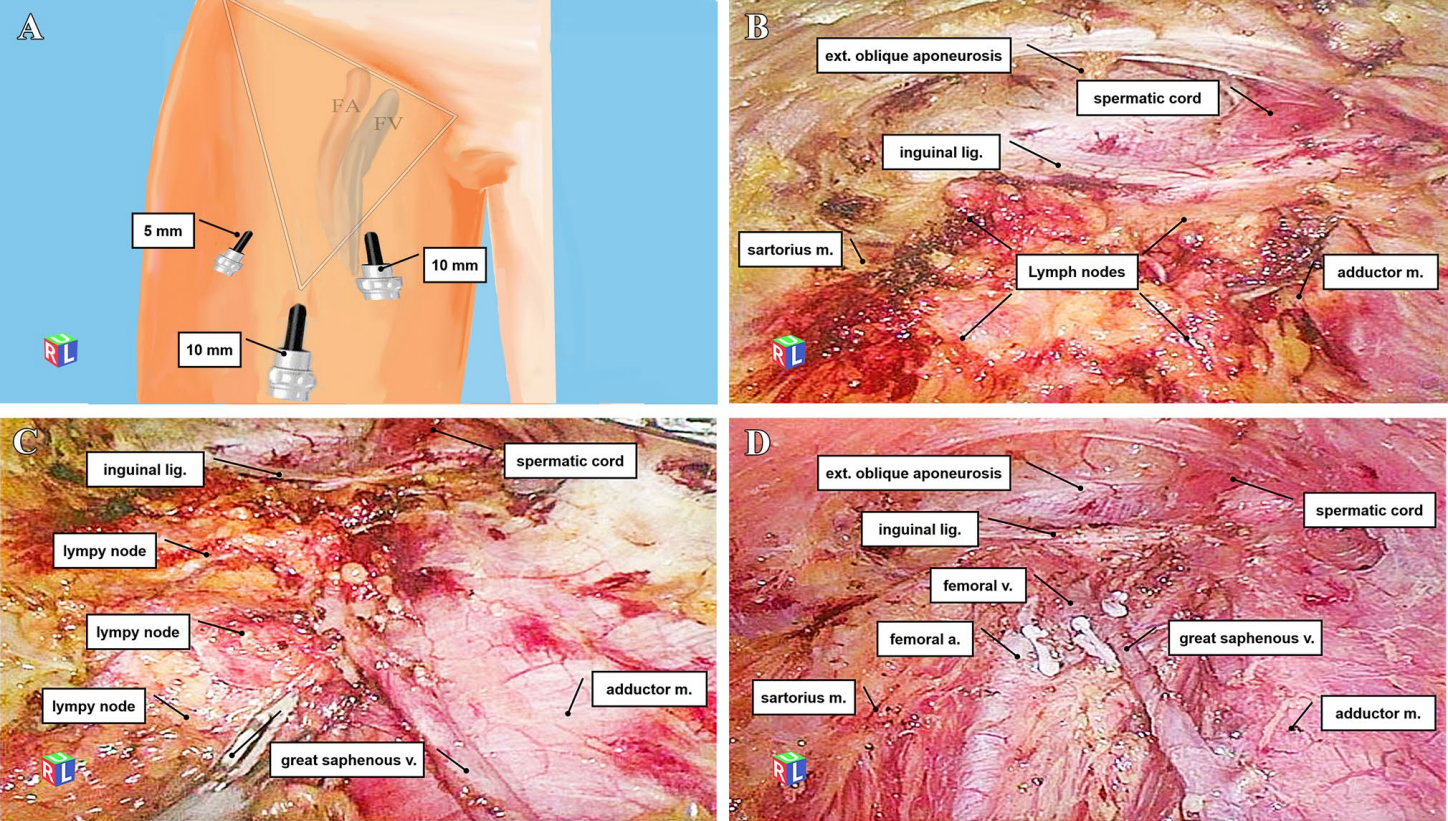
Endoscopic inguinal lymphadenectomy (right side). **A** Positions of trocars of endoscopic inguinal lymphadenectomy. **B** After creating “camping tent”-like working space, superficial and deep lymph nodes are successively dissected. **C** Removal of lymph nodes superiorly along the great saphenous vein. **D** View after radical lymphadenectomy, superficial and deep lymph nodes are removed, with sparing the great saphenous vein

Prophylactic EIL is recommended because many patients originate from remote rural areas in China where access to healthcare is limited. Prophylactic resection proves advantageous for these individuals. The techniques for EIL, including incision placements and the number of trocars used, were consistent with previously documented literature [4] (Fig. 3A). Creating a working space for inguinal lymph node dissection using two-dimensional laparoscopy is crucial. The anterior operational area took on the appearance of a “camping tent.” The upper section of this tent-like space consists of the skin and subcutaneous tissue, playing a pivotal role in maintaining continuous lymphatic and vascular supply to the overlying skin. In contrast, the lower portion encompasses deeper tissues, including superficial inguinal lymphatic tissues, the great saphenous vein, fascia lata, and thigh muscles (Fig. 3B). The boundaries for EIL dissection align with those for OIL, with particular attention to the great saphenous vein as an anatomical reference point (Fig. 3C). Once the saphenofemoral junction was exposed, meticulous dissection and control of the entrance of the long saphenous vein were conducted using polymer clips. Whenever feasible, efforts were made to preserve the saphenous vein itself and its branches, without compromising the dissection. Subsequently, the anterior surface of the femoral vein was cleared, and the inguinal ligament was identified by its silver-white transverse fibers. An ultrasonic scalpel was employed to open the femoral sheath, facilitating dissection of the femoral artery, followed by clearance of the femoral vein medial to it. Both vessels are meticulously skeletonized. The deep inguinal lymph nodes, also known as Cloquet nodes, were dissected from the inguinal ligament to the fossa ovalis and marked with clips for pathological analysis. Caudal dissection continued along the femoral and saphenous tracts. All lymphatic tissue and nodes, both superficial and deep within the region, were completely resected *en bloc*. After completing the inguinal region dissection, the saphenous vein (if preserved), femoral vein and artery, as well as the pectineus, adductor longus, and sartorius muscles, were clearly identified (Fig. 3D). To assess lower extremity edema, measurements of thigh perimeter were taken, and a suction drainage system was inserted through the lateral incision. A light pressure dressing was applied over the groin region until the patient was discharged from the hospital.

### Assessment of complications

In our study, the intraoperative adverse events were collected and reported according to the ICARUS Global Collaboration Checklist [9]. The assessment of these intraoperative adverse events was carried out using a well-established severity grading system as previously detailed [10]. For the evaluation of postoperative complications, we closely followed the comprehensive guidelines presented in the EAU proposal [11]. The statistical analysis was conducted utilizing the Clavien–Dindo classification system, with special attention paid to situations in which multiple complications were encountered by a single patient. Grade I complications were categorized according to the highest observed Clavien–Dindo grade within each patient.

### Postoperative care

Following the surgical procedure, the groin region was securely bandaged with elastic material, and this dressing remained in place until the patient’s discharge from the hospital. To monitor and assess the presence of lower extremity edema, measurements of the thigh perimeter were regularly taken. Pain management was achieved through oral medications, and patients were encouraged to adhere to a diet characterized by low-fat and high-protein content to support their recovery. The drainage tube remained in place until the drainage output consistently stayed below 15 mL per day for 2 consecutive days or until the patient could ambulate effectively.

### Statistical analysis

Statistical analysis was performed using SPSS 27.0 software. Numerical variables were compared using *t*-test, while categorical variables were compared using the χ^2^ test and Fisher’s exact test. Survival analysis was performed using the Kaplan–Meier test. A *P*-value of less than 0.05 was considered statistically significant.

## Results

The demographic and clinicopathologic data for both groups are summarized in Table 1. No statistically significant differences were found between the two groups in terms of age, BMI, tumor size, and stage (*P* > 0.05). Notably, the EIL group exhibited a significantly longer duration of surgery compared to the OIL group (80.2 ± 4.3 min vs 53.1 ± 2.2 min, *P* = 0.00). Conversely, the EIL group experienced significantly lower blood loss compared to the OIL group (27.3 ± 1.5 ml vs 50.0 ± 2.7 ml, *P* = 0.00). Additionally, the OIL group had longer drainage periods and hospital stays in comparison to the EIL group (*P* < 0.01 and *P* = 0.00, respectively). In both groups, there were three cases of inguinal metastases among non-palpable lymph node patients (3/18, 16.7% vs 3/19, 15.8%), and no significant differences were observed in the total number of lymph nodes removed or the number of positive lymph nodes (*P* = 0.78).

Table 2 details the perioperative complications in both groups. One patient in the EIL group, characterized by a low BMI, experienced hypercarbia and pneumoderm during the right lymphadenectomy (Grade 1), necessitating hyperventilation and adequate fluid transfusion. Fortunately, no postoperative consequences were reported, and it’s worth mentioning that no intraoperative adverse events occurred in the OIL group (*P* = 0.03). The overall complication rates in the OIL and EIL groups were notably distinct, with 73.91% (17/23) in the OIL group and 25.92% (7/27) in the EIL group, demonstrating statistical significance (*P* = 0.00). When considering seroma and lymphocele, no significant differences were observed (*P* = 1.00). However, the OIL group exhibited significantly higher rates of wound infection, necrosis, lymphorrhagia, and lower extremity edema in comparison to the EIL group (*P* < 0.05). Comparative analysis of grade I and II complications indicated no significant differences between the two groups (*P* > 0.05). Notably, a statistically significant difference was identified in the occurrence of grade III complications (*P* < 0.05). Importantly, it is worth noting that neither group experienced grade IV or grade V complications (Table 3).

**Table 2:** Comparison of postoperative complications of the two groups

Complications	OIL (n = 23)	EIL (n = 27)	*χ*^2^	*P*
Incision infection & dehiscence	4	0	1.82	0.01
Incision necrosis	4	0	1.82	0.01
seroma	2	2	0.00	1.00
lymphorrhagia	2	0	2.22	0.02
Lymphocele	2	2	0.00	1.00
Lower extremity oedema	2	1	0.00	1.00
Hypercarbia	0	1	1.05	0.03

**Table 3:** Postoperative complications of Clavien–Dindo classification in the two groups (*n*/%)

Clavien–Dindo classification	OIL (*n* = 23)	EIL (*n* = 27)	*χ*^2^	*P*
I	5 (21.74)	5 (18.51)	0.08	1.00
II	2 (7.40)	2 (3.70)	0.03	0.87
IIIa	10 (26.08)	0 (7.40)	14.67	0.01
IIIb	0	0	0.00	0.00
IV	0	0	0.00	0.00
V	0	0	0.00	0.00

**Fig. 4:**
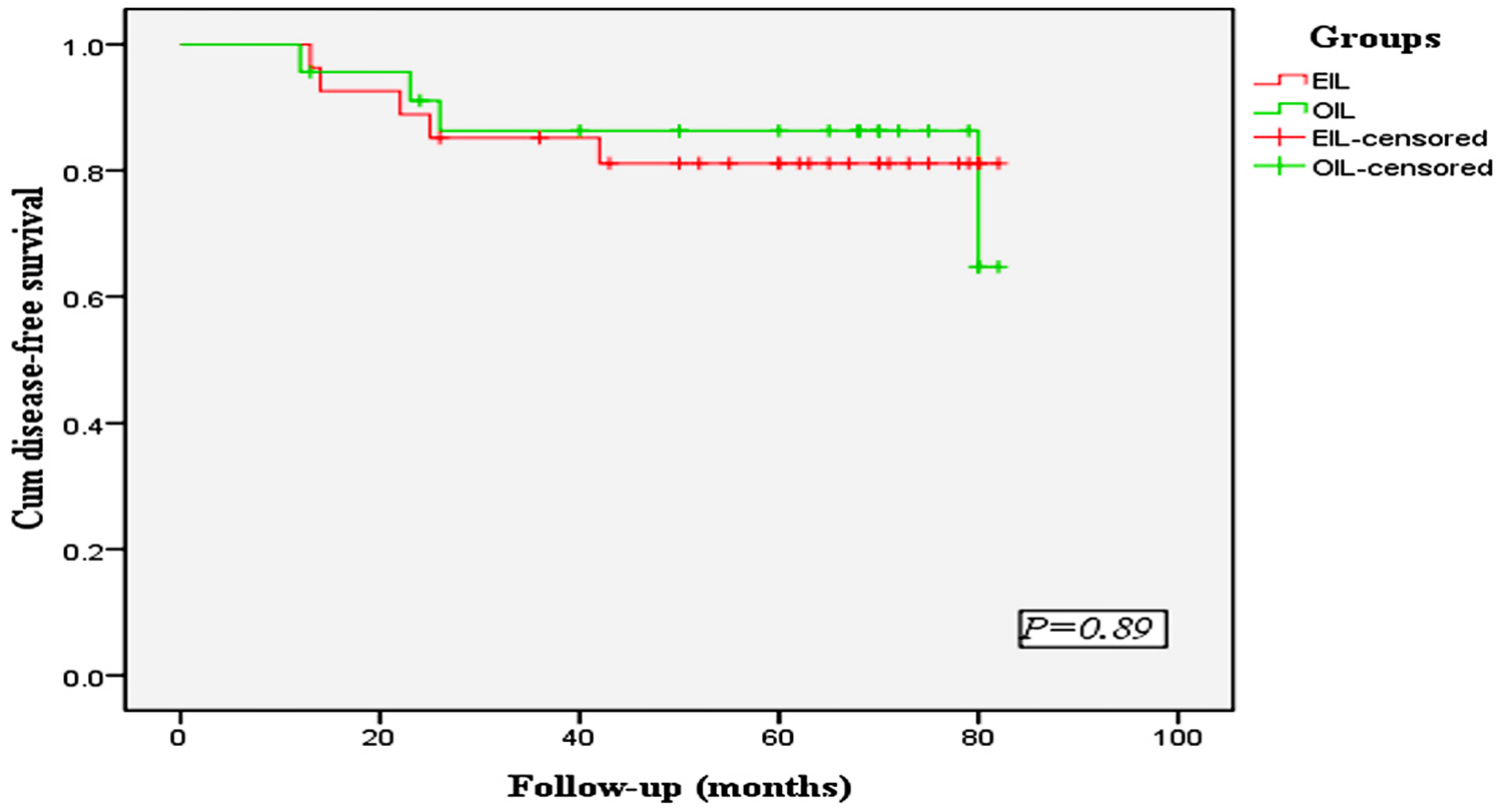
The 5-year overall survival rate of patients in the two groups

Follow-up interviews were conducted in person or via telephone. The median follow-up period was 60.4 months for the OIL group and 59.8 months for the EIL group (*P* = 0.45). Four patients in the OIL group who were staged II died of tumor recurrence within 3 years after surgery, while 6 patients in the EIL group who were staged II died, five of whom died of recurrence and one died of a heart attack 4 years after surgery. The 5-year disease-specific survival rates were 73.2% in the OIL group and 71.0% in the EIL group, with no significant difference observed between the two groups (*P* = 0.89) (Fig. 4).

## Discussion

Penile cancer is predominantly squamous cell carcinoma, with lymphatic drainage following the venous drainage system [12–15]. The primary route of metastasis is through the superficial inguinal pathway, followed by the deep inguinal lymphatic basin and external iliac nodes [14, 15]. Penile cancer has a long local regional period before metastasis to distant sites, which offers an opportunity to cure locally advanced penile cancer through the removal of involved regional nodes [16]. However, preoperative evaluation is necessary to determine which patients are most likely to have metastases in regional nodes and to select the optimal timing for surgery. Studies have reported that 20–25% of patients with penile carcinoma present with lymph node metastasis at the time of diagnosis, and the incidence of nodal metastases increases with tumor classification [14, 17–24]. Bilateral inguinal lymphadenectomy is currently accepted as a prognostic and therapeutic procedure in cases of penile cancer with a high risk of developing metastasis. Complete early bilateral lymphadenectomy offers the best chance of cure in patients with nodal disease since lymphatic drainage is often bilateral [4, 14, 17, 19–26].

This study aimed to compare the efficacy and safety of two surgical approaches in patients with penile cancer at T1 and T2 stages. The primary outcome was cancer recurrence, while the secondary outcomes included various postoperative parameters, such as blood loss, wound infection, necrosis, lymphorrhagia, lower extremity edema, drainage, hospital stay, and operation time. The results demonstrated that both surgical approaches were equally effective in preventing cancer recurrence, consistent with previous research that has found no significant difference between the two methods. Moreover, the study findings revealed that there was no significant difference in the number and positivity of lymph nodes removed between the two groups. The average number of lymph nodes harvested in the EIL group was 11.1 per leg, which was similar to that in the open surgery group (11.6 per leg) [6, 14]. These results suggest that both surgical approaches were equally successful in detecting and removing lymph nodes, which is a critical aspect of precise staging and identifying the appropriate treatment. The node count is regarded as an important measure of the quality of lymph node dissection in endoscopic procedures [4]. In our series, up to 16% of clinically node negative (cN0) inguinal basins exhibited occult metastases, which is in line with prior reports in the literature [15–17]. We believe that early prophylactic inguinal lymphadenectomy was beneficial for these patients, despite the challenge in treating those with non-palpable inguinal nodes. Based on these findings, we are confident that the endoscopic procedure adheres to the principles of the conventional technique, which aims to achieve radical resection of inguinal lymph nodes. Thus, the choice of surgical approach may depend on surgeon preference, experience, and resource availability.

However, the secondary outcomes differed significantly between the two groups, with the EIL group showing a reduction in blood loss, wound infection, necrosis, lymphorrhagia, lower extremity edema, drainage, and hospital stay compared to the OIL group. The findings of this study are consistent with the established advantages of minimally invasive surgery, which have been demonstrated to decrease the incidence of postoperative complications, pain, and recovery time. The endoscopic approach used in this study provided several advantages over traditional open surgery. First, the technique involved the use of three smaller incisions, which minimized damage to the skin and subcutaneous tissue and reduced the risk of mechanical retraction damage. Second, the dissection of the flap was performed in a specific anatomic plane, which ensured that the lymphatic and blood supply to the flap was preserved and thus less likely to cause serious complications such as ischemic necrosis of the skin [4]. Third, appropriate ligation of lymphatic channels using a harmonic scalpel, coagulator, or clips was critical in reducing morbidity associated with the lymphatic system. Lastly, this technique was successfully used in obese patients, who are often challenging to operate on using open surgery. However, it is important to note that EIL surgery had a significantly longer operation time compared to OIL due to the technical complexity of endoscopic surgery.

Several limitations of this study should be considered when interpreting the results, such as its retrospective nature, which may introduce biases and confounding factors that were not controlled for. Additionally, the sample size was relatively small, which may limit the generalizability of the findings. Lastly, the follow-up period was relatively short and may not capture long-term outcomes such as overall survival and quality of life.

## Conclusions

In conclusion, the study provides evidence that both OIL and EIL are effective in preventing cancer recurrence in patients with T1 and T2 stage penile cancer. Despite no significant difference in the primary outcome of cancer recurrence between the two surgical approaches, the EIL group had significantly fewer postoperative complications and longer operation time than the OIL group. Hence, the choice of surgical approach may depend on the surgeon’s experience, preference, and resource availability. Further studies with larger sample sizes and longer follow-up periods are needed to validate the findings and determine the long-term outcomes of the two surgical approaches.

## References

[1] Gkegkes ID, Minis EE, Lavazzo C (2018). Robotic-assisted inguinal lymphadenectomy: a systematic review. J Robot Surg.

[2] Yang M, Liu Z, Tan Q, Hu X, Liu Y, Wei L, Deng C, Zhou S, Yang N, Duan G, Zheng Y, Li X, Chen Z, Zhou Z, Zheng J (2023). Comparison of antegrade robotic assisted VS laparoscopic inguinal lymphadenectomy for penile cancer. BMC Surg.

[3] Yao K, Tu H, Li YH, Qin ZK, Liu ZW, Zhou FJ, Han H (2010). Modified technique of radical inguinal lymphadenectomy for penile carcinoma: morbidity and outcome. J Urol.

[4] Zhou XL, Zhan JF, Zhan JF, Zhou SJ, Yuan XQ (2013). Endoscopic inguinal lymphadenectomy for penile carcinoma and genital malignancy: a preliminary report. J Endourol.

[5] Lopes A, Rossi BM, Fonseca FP, Morini S (1996). Unreliability of modified inguinal lymphadenectomy for clinical staging of penile carcinoma. Cancer.

[6] Korkes F, Moniz RR, Castro MG, Guidoni LRM, Fernanders RC, Perez MDC (2009). Modified inguinal lymphadenectomy for penile carcinoma has no advantages. J Androl Sci.

[7] Tobias-Machado M, Tavares A, Molina WR, Zambon JP, Forsetto P, Juliano RN, Wroclawski ER (2005). Comparative study between video endoscopic inguinal lymphadenectomy (VEIL) and standard open procedure for penile cancer: preliminary surgical and oncological results [abstract]. J Urol.

[8] Elsamra SE, Poch MA (2017). Robotic inguinal lymphadenectomy for penile cancer: the why, how, and what. Transl Androl Urol.

[9] Cacciamani GE, Sholklapper T, Dell’Oglio P, Rocco B, Annino F, Antonelli A (2022). The Intraoperative Complications Assessment and Reporting with Universal Standards (ICARUS) global surgical collaboration project: development of criteria for reporting adverse events during surgical procedures and evaluating their impact on the postoperative course. Eur Urol Focus.

[10] Biyani CS, Pecanka J, Rouprêt M, Jensen JB, Mitropoulos D (2020). Intraoperative adverse incident classification (EAUiaiC) by the European Association of Urology ad hoc Complications guidelines panel. Eur Urol.

[11] Mitropoulos D, Artibani W, Graefen M, Remzi M, Rouprêt M, Truss M (2011). Reporting and grading of complications after urologic surgical procedures: an ad hoc EAU guidelines panel assessment and recommendations. Eur Urol.

[12] Sanchez DF, Fernandez-Nestosa MJ, Cañete-Portillo S, Cubilla AL (2022). Evolving insights into penile cancer pathology and the eighth edition of the AJCC TNM staging system. Urol Oncol.

[13] Ahmed ME, Khalil MI, Kamel MH, Karnes RJ, Spiess PE (2020). Progress on management of penile cancer in 2020. Curr Treat Opt Oncol.

[14] Swan MC, Furniss D, Cassell OC (2004). Surgical management of metastatic inguinal lymphadenopathy. BMJ.

[15] de Carvalho JP, Patrício BF, Medeiros J, Sampaio FJ, Favorito LA (2011). Anatomic aspects of inguinal lymph nodes applied to lymphadenectomy in penile cancer. Adv Urol.

[16] Johnson TV, Hsiao W, Delman KA, Jani AB, Brawley OW, Master VA (2010). Extensive inguinal lymphadenectomy improves overall 5-year survival in penile cancer patients: results from the Surveillance, Epidemiology, and End Results program. Cancer.

[17] Nelson BA, Cookson MS, Smith JA, Chang SS (2004). Complications of inguinal and pelvic lymphadenectomy for squamous cell carcinoma of the penis: a contemporary series. J Urol.

[18] Ornellas AA, Seixas AL, Marota A, Wisnescky A, Campos F, de Moraes JR (1994). Surgical treatment of invasive squamous cell carcinoma of the penis: retrospective analysis of 350 cases. J Urol.

[19] Stecca CE, Alt M, Jiang DM, Chung P, Crook JM, Kulkarni GS, Sridhar SS (2021). Recent advances in the management of penile cancer: a contemporary review of the literature. Oncol Ther.

[20] Aita GA, Zequi SC, Costa WH, Guimarães GC, Soares FA, Giuliangelis TS (2016). Tumor histologic grade is the most important prognostic factor in patients with penile cancer and clinically negative lymph nodes not submitted to regional lymphadenectomy. Int Braz J Urol.

[21] Diorio GJ, Leone AR, Spiess PE (2016). Management of penile cancer. Urology.

[22] Koifman L, Hampl D, Koifman N, Vides AJ, Ornellas AA (2013). Radical open inguinal lymphadenectomy for penile carcinoma: surgical technique, early complications and late outcomes. J Urol.

[23] Azevedo RA, Roxo AC, Alvares SHB, Baptista DP, Favorito LA (2021). Use of flaps in inguinal lymphadenectomy in metastatic penile cancer. Int Braz J Urol.

[24] Chahoud J, Kohli M, Spiess PE (2021). Management of advanced penile cancer. Mayo Clin Proc.

[25] Bevan-Thomas R, Slaton JW, Pettaway CA (2002). Contemporary morbidity from lymphadenectomy for penile squamous cell carcinoma: the M.D. Anderson Cancer Center Experience. J Urol.

[26] Delacroix SE, Pettaway CA (2010). Therapeutic strategies for advanced penile carcinoma. Curr Opin Support Palliat Care.

